# Dynamic Predictive Models with Visualized Machine Learning for Assessing the Risk of Lung Metastasis in Kidney Cancer Patients

**DOI:** 10.1155/2022/5798602

**Published:** 2022-10-14

**Authors:** Chan Xu, Qian Zhou, Wencai Liu, Wenle Li, Shengtao Dong, Wanying Li, Xiaofeng Xu, Ximin Qiao, Youli Jiang, Jingfang Chen, Chengliang Yin

**Affiliations:** ^1^Clinical Medical Research Center, Xianyang Central Hospital, Xianyang, China; ^2^Department of Respiratory and Critical Care Medicine, The First People's Hospital of Chong Qing Liang Jiang New Area, Chongqing, China; ^3^Department of Orthopaedic Surgery, The First Affiliated Hospital of Nanchang University, Nanchang, China; ^4^Xiamen University, Molecular Imaging and Translational Medicine Research Center, State Key Laboratory of Molecular Vaccinology and Molecular Diagnostics, Xiamen, China; ^5^Department of Spine Surgery, Second Affiliated Hospital of Dalian Medical University, Dalian 116000, China; ^6^Department of Urology, Xianyang Central Hospital, Xianyang, China; ^7^Hengyang Medical School, School of Nursing, University of South China, Hengyang, Hunan, China; ^8^National Clinical Research Center for Infectious Diseases, The Third People's Hospital of Shenzhen, Shenzhen, Guangdong, China; ^9^Faculty of Medicine, Macau University of Science and Technology, Macau, China

## Abstract

**Objective:**

To establish and verify the clinical prediction model of lung metastasis in renal cancer patients.

**Method:**

Kidney cancer patients from January 1, 2010, to December 31, 2017, in the SEER database were enrolled in this study. In the first section, LASSO method was adopted to select variables. Independent influencing factors were identified after multivariate logistic regression analysis. In the second section, machine learning (ML) algorithms were implemented to establish models and 10-foldcross-validation was used to train the models. Finally, receiver operating characteristic curves, probability density functions, and clinical utility curve were applied to estimate model's performance. The final model was shown by a website calculator.

**Result:**

Lung metastasis was confirmed in 7.43% (3171 out of 42650) of study population. In multivariate logistic regression, bone metastasis, brain metastasis, grade, liver metastasis, N stage, *T* stage, and tumor size were independent risk factors of lung metastasis in renal cancer patients. Primary site and sequence number were independent protection factors of LM in renal cancer patients. The above 9 impact factors were used to develop the prediction models, which included random forest (RF), naive Bayes classifier (NBC), decision tree (DT), xgboost (XGB), gradient boosting machine (GBM), and logistic regression (LR). In 10-foldcross-validation, the average area under curve (AUC) ranked from 0.907 to 0.934. In ROC curve analysis, AUC ranged from 0.879–0.922. We found that the XGB model performed best, and a Web-based calculator was done according to XGB model.

**Conclusion:**

This study provided preliminary evidence that the ML algorithm can be used to predict lung metastases in patients with kidney cancer. This low cost, noninvasive and easy to implement diagnostic method is useful for clinical work. Of course this model still needs to undergo more real-world validation.

## 1. Introduction

Kidney cancer, accounting for 5% of all cancers, originates from the renal tubular and collecting tubular epithelial system [1]. The incidence trend has been gradually increasing in recent years, resulting in a huge medical burden. The prevalence rate of men is approximately twice that of women [1]. Additionally, obesity, diabetes, hypertension, smoking, kidney injury, and drugs are major risk factors of kidney cancer. The principal manifestations of kidney cancer were hematuria, renal pain, and mass [2, 3]. In the early stage of the disease, the symptoms are not noticeable. As the result, when patients intend to seek a healing care, they may have been in a metastatic state of kidney cancer and are suffering from the corresponding complications. The five-year survival rates of stage I and II were about 88% to 95%, and cancer-specific survival (CSS) rates were 84% to 95% [4]. Renal cell carcinoma (RCC), making up 90% of kidney cancer, is the sixth and eighth most common cancer among American men and women in 2021 [5]. RCC is mainly composed of clear-cell RCC, papillary RCC, and chromophobe RCC [6, 7]. Renal clear cell carcinoma, accounts for about 70% of RCC, is invasive and has a poor prognosis. The survival time of renal clear cell carcinoma is from 3 months to 5 years. 60% of these patients die within 1 to 2 years after diagnosis [5, 8–11].

Metastasis from kidney cancer is not rare. Highly vascularization can lead to local progression and increase the chance of distant spread [6]. There have been relevant studies on the occurrence, development, and metastasis. Hypoxia-irreducible factor (HIF) and epithelial-mesenchymal transition (EMT) and so on are important molecular events [6, 11]. Nishida et al. indicated that amplification of cancer-cell-intrinsic inflammation can trigger neutrophil-dependent lung metastasis during RCC progression [8]. Lung and bone are common metastatic sites of kidney cancer [12]. At the time of initial diagnosis, 18%–40% of patients have already developed systemic metastases. In addition, metastasis is widespread in the long-termfollow-up after nephrectomy [4, 7, 9, 13, 14]. The study of Jianxin Xue and colleagues reported that 2931 of 33449 RCC had distant metastasis and lung (6.19%) was the most common site of metastasis [7]. Pulmonary metastases are multiple nodules with bilateral distribution or solitary masses. The lower lobes of the lung were common sites. Immune checkpoint inhibitors (ICI), antiprogrammed death-1 (PD-1) antibody, and anticytotoxic T lymphocyte-associated antigen 4 (CTLA-4) antibody were accepted as treatments for metastatic RCC [15]. However, the survival rate of metastatic kidney cancer is just about 20% [6].

Clinical models of kidney cancer have been established, but the main focus is to predict the prognosis. The UCLA (University of California, Los Angeles) integrated staging system (UISS) and the risk model of the International Metastatic RCC Database Consortium (IMDC) are examples [16]. Machine learning is a subfield of artificial intelligence. It has many applications in kidney cancer such as identifying pathological variants, grading judgments, and differentiating benign from malignant renal tumors [17].

At present, there are few reports of machine learning model to predict lung metastasis of kidney cancer. In this study, we collected data from the SEER database to establish models. After checking performance of model, a Web calculator was conducted to assist clinicians in predicting lung metastasis from kidney cancer.

## 2. Methods

### 2.1. Patients' Populations

Patients with kidney cancer from January 1, 2010, to December 31, 2017, in the SEER database were enrolled in this study. The inclusion criteria were listed as follows: (1) patients definitely diagnosed as primary kidney cancer when they were alive with ICD-O (International Classification of Diseases for Oncology) of 8120/3, 8130/3, 8260/3, 8310/3, 8312/3, and 8317/3; (2) histological subtypes of kidney cancer were clear cell RCC, papillary, chromophobe, and any others. The exclusion criteria were listed as follows: (1) age of patients was younger than 18; (2) patients with other primary tumors at diagnosis; and (3) the clinicopathological results were uncompleted.

### 2.2. Data Collections

Marital, age, race, sequence number, survival time, status, sex, primary site, grade, laterality, pathological, *T* stage, N stage, tumor size, bone metastasis, brain metastasis, liver metastasis, and lung metastasis were collected retrospectively. Data were extracted from the SEER database with the help of SEER^*∗*^STAT software 85 (version 8.3.5). The process of extraction was carried out by two independent data collectors. If there was any disagreement, a third collector would bring in to assist with the final decision.

### 2.3. Statistical Methods

Mean was used to describe continuous variables following a normal distribution. Numerical values and proportions were used to describe categorical variables. We concluded a comparison between groups using chi-squared tests, *t*-tests, and logistic regression analysis. Variables with nonzero coefficients in the least absolute shrinkage and selection operator (LASSO) analysis were chosen for further analysis. Variables with *p* < 0.05 in univariate logistic regression analysis were put into multivariate logistic regression analysis. Independent risk factors were determined after multivariate logistic regression analysis. ML algorithms, such as RF, NBC, DT, XGB, GBM, and LR, were implemented to establish models. We ranked the importance of the variables for each model. XGB is an integration algorithm based on boost. It is typical of the integration of cart tree, which is an improvement of the gradient tree boosting.

(1) $$\begin{array}{c} ℒ\left(\emptyset \right)=\sum_{i}l\left({\hat{y}}_{i},y_{i}\right)+\sum_{k}\Omega \left(f_{k}\right)\;,\;\text{where }\Omega \left(f\right)=\;rT+\;\frac{1}{2}\lambda \omega^{2}. \end{array}$$

Here, *l* is a differentiable convex loss function that measures the difference between the prediction ^ *yi* and the target *yi*. The second term Ω penalizes the complexity of the model. The probabilistic output results are evaluated using receiver operating characteristic curve (ROC). 10-foldcross-validation and ROC curve analysis were conducted to evaluate the performance of models. Maximum AUC was the basis for determining the best model. Heatmap showed the correlation between various variables in the models. The number in each grid of heatmap represented the correlation coefficient, and the color depth was negatively correlated with the correlation of variables. According to the results of the best model, a Web calculator was established.

## 3. Results

### 3.1. Basic Characteristics

A total of 42650 kidney cancer patients from the SEER database were enrolled in this study. A total of married was 25058 (58.75%) with a median age of 64.000 [55.000, 73.000]. Marital, race, primary site, grade, laterality, pathological, *T* stage, N stage, bone metastasis, and liver metastasis were variables with statistically significant differences (*p* < 0.05). White male was the main population.

As shown in Table 1, there were 3171 kidney cancer patients with lung metastasis and 39479 kidney cancer patients without lung metastases. Through comparing data of the two groups above, we obtained the result that the differences of all variables were statistically significant (*p* < 0.05).

### 3.2. Independent Risk Factor Selection

**Figure 1:**
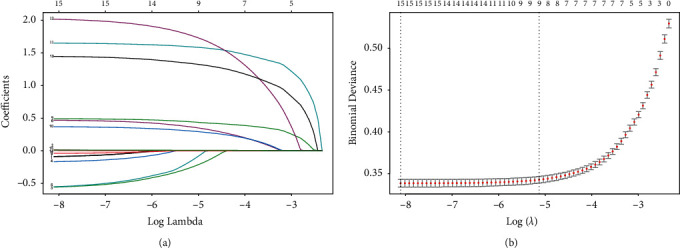
Variable selection using LASSO method. (a) A coefficient profile plot. The vertical axis represents the coefficients, and the horizontal axis represents log (lambda). (b) A binomial deviance curve. The vertical axis represents the binomial deviance, and the horizontal axis represents log (lambda). Vertical lines were drawn based on 1 standard error criteria. 9 variables with nonzero coefficients were selected by optimal lambda.

**Figure 2:**
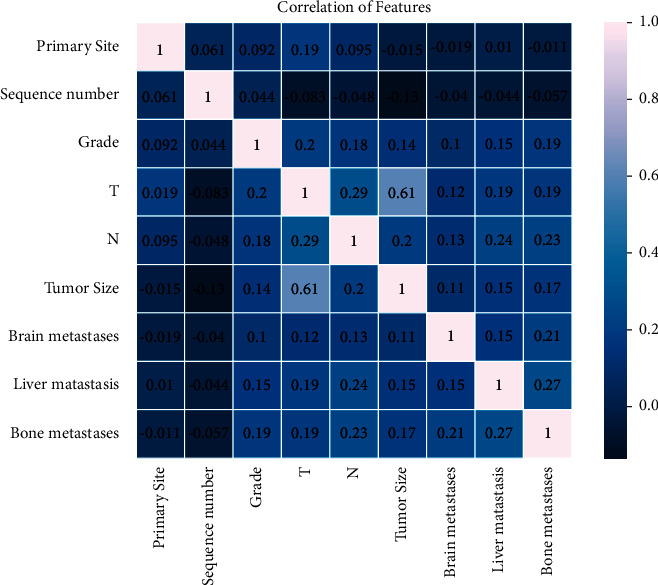
Heatmap analysis of nine variables.

As shown in Figure 1, nine variables with nonzero coefficients in LASSO analysis were selected for logistic regression. As shown in Table 2, bone metastasis, brain metastasis, grade, liver metastasis, N stage, primary site, sequence number, *T* stage, and tumor size were factors with *p* < 0.05 in univariate logistic regression analysis. After multivariate regression analysis, we identified that bone metastasis (yes, OR = 4.83, 95% CI = 4.27–5.46, *p* < 0.001), brain metastasis (yes, OR = 8.41, 95% CI = 6.72–10.51, *p* < 0.001; unknown, OR = 6.13, 95% CI = 2.35–15.98, *p* < 0.001), grade (poorly differentiated, OR = 2.71, 95% CI = 1.82–4.04, *p* < 0.001; undifferentiated; anaplastic, OR = 4.58, 95% CI = 3.05–6.87, *p* < 0.001; unknown, OR = 6.34, 95% CI = 4.29–9.37, *p* < 0.001), liver metastasis (yes, OR = 4.23, 95% CI = 3.6–4.96, *p* < 0.001; unknown, OR = 6.36, 95% CI = 3.06–13.21, *p* < 0.001), N stage (*N*1, OR = 3.79, 95% CI = 3.37–4.25, *p* < 0.001; *N*2, OR = 3.54, 95% CI = 2.21–5.69, *p* < 0.001; *N*X, OR = 2.33, 95% CI = 1.96–2.77, *p* < 0.001), *T* stage (T2, OR = 3.42, 95% CI = 2.93–4, *p* < 0.001; T3, OR = 4.44, 95% CI = 3.89–5.07, *p* < 0.001; T4, OR = 5.39, 95% CI = 4.42–6.57, *p* < 0.001; TX, OR = 5.67, 95% CI = 4.63–6.94, *p* < 0.001), and tumor size (OR = 1.01, 95% CI = 1–1.01, *p* < 0.001) were independent risk factors of LM in renal cancer patients. Furthermore, we found that primary site (C65.9-Renal pelvis, OR = 0.38, 95% CI = 0.3–0.49, *p* < 0.001) and sequence number (more, OR = 0.62, 95% CI = 0.56–0.69, *p* < 0.001) were independent protection factors. As shown in Figure 2, each grid in the heatmap visually showed the correlation coefficient between each variable with color depth.

### 3.3. Development and Validation of Predictive Models

**Figure 3:**
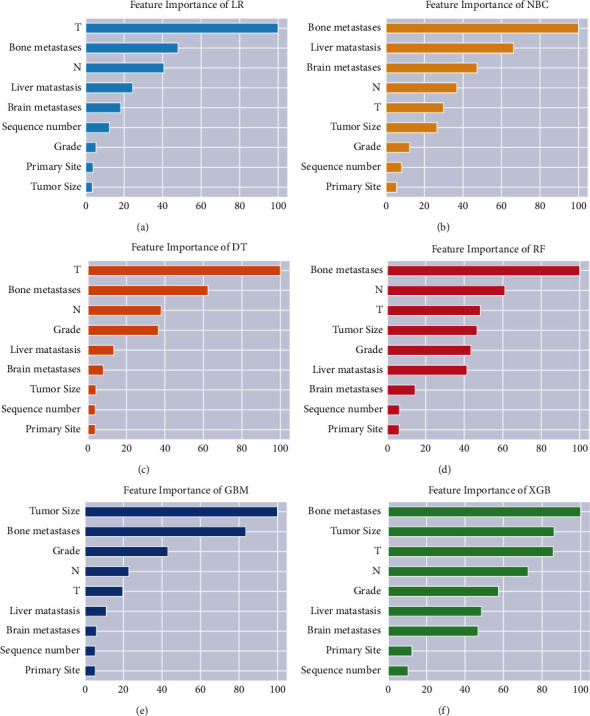
Relative importance ranking of each input variable for 6 predicting models.

**Figure 4:**
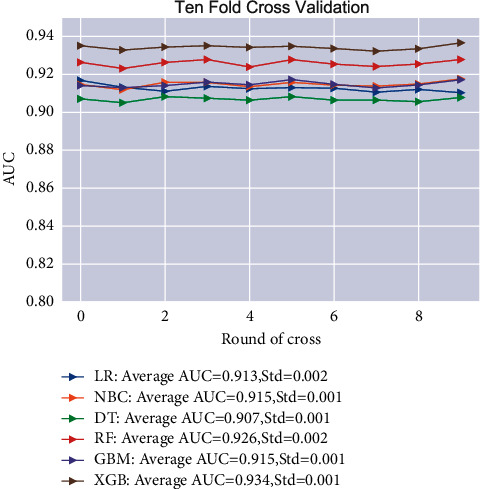
10-foldcross-validation of 6 ML algorithms for predicting lung metastasis in patients with renal cancer.

For developing ML models, nine independent predictors, with *p* < 0.05 in the multivariate regression analysis, were used for model establishment. And lung metastasis status was also included as the outcome index in the models.  Figure 3 demonstrated the relative importance ranking of each input variable in the models. The ranking of variables in each model was very different. The patients with bone metastasis and the *T* stage were variables with relatively high importance ranking in all models. However, primary site and sequence number were variables with relatively low importance ranking in all models. For the XGB, the relative importance rank of all variables from high to low was bone metastasis, tumor size, *T* Stage, *N* stage, grade, liver metastasis, brain metastasis, primary site, and sequence number. We applied ML algorithms such as RF, NBC, DT, XGB, GBM, and LR to establish models. The results of 10-foldcross-validation (Figure 4) show that the average AUC of all models was above 0.9. And all six ML models fitted well during the course of ten iterations. The XGB's average AUC was 0.934 (std = 0.001). As a result, XGB model was selected as the final prediction model.

### 3.4. Web-Based Calculator

**Figure 5:**
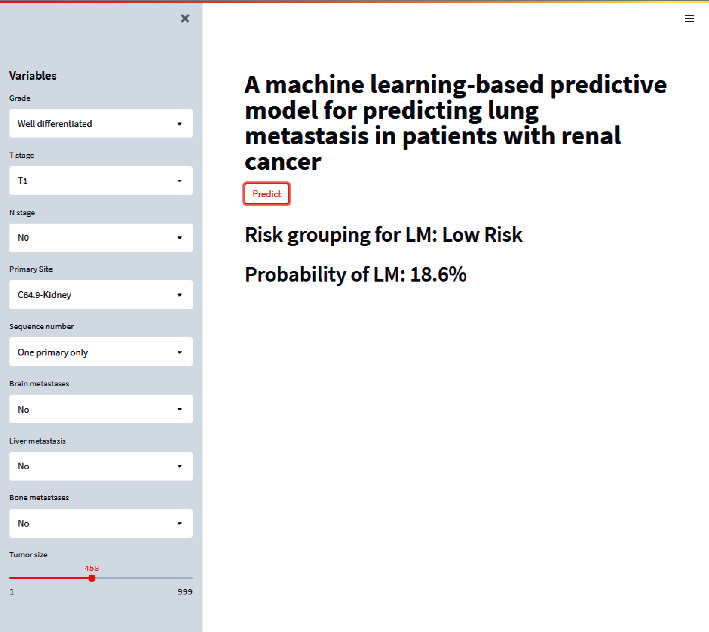
An example of the online calculator for predicting lung metastasis in renal cancer.

In order to facilitate clinical application, a Web-based calculator was established on the basis of XGB model (https://share.streamlit.io/liuwencai4/renal_lung/main/renal_lung.py). As shown in Figure 5, users can input values of each variable through clicking and selecting. Risk grouping for LM and probability of LM in renal cancer will be showed.

## 4. Discussion

Lung is the most common metastatic site of kidney cancer [7]. Early diagnosis of metastasis can improve the feasibility of surgery and increase the survive chance. The profile of kidney cancer patients is complex and involves multidisciplinary treatment issues. Artificial intelligence can be well applied in this field because of its powerful information extraction and processing ability [16]. Therefore, this study aimed to develop a highly accurate model capable of predicting lung metastasis from kidney cancer.

We identified nine influence factors, included bone metastasis, brain metastasis, grade, liver metastasis, *T* stage, N stage, primary site, sequence number, and tumor size. In addition, 10-foldcross-validation was adopted to check the performance of models. Finally, the model with the highest accuracy is presented as a Web calculator for application.

Our study found that organ metastases were important influencing factors. Many patients will develop multiple organ metastases. In the study of Wei Xi, metastases of two or more sites accounted for 33% [18]. Jianxin Xue's study also found that there were 8.76% patients with clear-cell RCC, which had distant metastases at the time of diagnosis, and 35.01% (1026/2931) metastatic patients had multiple metastases [7]. This finding was consistent with the results of the present study. Furthermore, organ metastases as predictors have also been reported in previous studies. Shengtao Dong et al. constructed a bone metastasis risk prediction model based on brain metastasis, liver metastasis, and lung metastasis as predictors [19]. Bone metastasis, liver metastasis, and brain metastasis were strong predictors in the models of our study.

As shown in Figure 3, important factors in constructing XGB, RF, and NBC models to predict lung metastasis from kidney cancer were prioritized.

Variables including *T* stage, *N* stage, and pathological grade were associated with the development of LM in renal cell carcinoma [20]. These risk factors were also important in other distant metastases of kidney cancer [1, 7]. This highlights the significance of the stage and grade in predicting renal cell carcinoma organ metastasis. In addition, N stage and *T* stage were used not only to predict kidney cancer metastasis but also as an important parameter in prognostic models. For example, the University of California School of Medicine used the stage to predict five years survival in metastatic and nonmetastatic patients [21].

Tumor size was an independent predictor of overall survival [4]. The pseudocapsule (PS) in kidney cancer is the fibrous interface between the tumor and renal parenchyma [22]. There is a richer blood supply system around PS. With PS from being infiltrated to penetrate, the incidence of venous tumor thrombus (VTT) and microvascular invasion (MVI) increases [23]. Thus, further distant metastasis occurs. The probability of distant metastasis may increase in the process of primary lesions expansion owing to an increase of PS surface area.

Our study revealed that primary site was a protection factor. In addition, the renal pelvic cancer was less likely to transfer to the lung than renal cancer. Because renal cancer originates from the epithelium of the proximal tubules, renal pelvic cancer originates from the urothelium. It is more likely to be diagnosed and treated in the early stage because of the high incidence of hematuria. Vascularization is an important condition for tumor growth, invasion, and metastasis [24]. The blood supply of renal pelvic cancer may be less than that of renal cancer [25]. Sequence number was another independent protection factor of LM in kidney cancer. We found that patients with >1 primary tumor were less likely to spread to lung. One of our guesses was that patients with multiple tumors may have insufficient time to form LM because of poor prognosis. Another explanation was that more symptoms could promote early diagnosis and medical treatment. The exact mechanism needs to be further explored.

Few studies have been performed to predict LM in patients with renal cell carcinoma. Although some studies have reported some biomarkers other than the above predictors for LM prediction [26], few of these markers have been applied. Previously, Xinyu Sheng's team at the Zhejiang University School predicted LM in kidney cancer patients based on patient data from the SEER database, with a column line plot of development and a model constructed based on TNM stages with ground AUC of 0.780 and 0.618, respectively, and the study was not externally validated [20, 27]. Although the AUC of the models developed in the training set is greater than 0.50, there is still room for improvement. However, the AUCs of the six models constructed in this study based on machine learning are all above 0.9, which reflects the good robustness of the models. We expect that the network calculator constructed with the XGB model in this study can be applied or tested in the future.

This study also had some limitations. First of all, the indicators including metastasis sites and some serological data in SEER database are not comprehensive [7, 12]. Secondly, further verification of multicenters is indeed in the future.

## 5. Conclusion

This study provided preliminary evidence that the ML algorithm can be used to predict lung metastases in patients with kidney cancer. However, the prediction model cannot specify the genetic characteristics of these patients. However, this low-cost, noninvasive, and easy to implement diagnostic method is useful for clinical work. Of course this model still needs to undergo more real-world validation.

## Figures and Tables

**Table 1:** Renal cancer with or without lung metastasis at baseline.

Characteristics	Level	No (*N* = 39479)	Yes (*N* = 3171)	*p*
Marital (%)	Married	23255 (58.90)	1803 (56.86)	<0.0001
	Unknown	2021 (5.12)	102 (3.22)	
	Unmarried	14203 (35.98)	1266 (39.92)	

Age (median [IQR])	NA	64.000 (55.000, 72.000)	65.000 (57.000, 75.000)	<0.0001

Race ethnicity (%)	Black	5109 (12.94)	280 (8.83)	<0.0001
	Chinese	471 (1.19)	41 (1.29)	
	Other	3114 (7.89)	291 (9.18)	
	White	30785 (77.98)	2559 (80.70)	

Sequence number (%)	More	13427 (34.01)	603 (19.02)	<0.0001
	One primary only	26052 (65.99)	2568 (80.98)	

Times (mean (SD))	NA	41.102 (30.576)	13.674 (18.325)	<0.0001

Status (%)	Alive	30809 (78.04)	692 (21.82)	<0.0001
	Dead	8670 (21.96)	2479 (78.18)	

Sex (%)	Female	14133 (35.80)	946 (29.83)	<0.0001
	Male	25346 (64.20)	2225 (70.17)	

Primary site (%)	C64.9-Kidney	37524 (95.05)	3042 (95.93)	0.0294
	C65.9-Renal pelvis	1955 (4.95)	129 (4.07)	

Grade (%)	Moderately differentiated	14413 (36.51)	238 (7.51)	<0.0001
	Poorly differentiated	8369 (21.20)	546 (17.22)	
	Undifferentiated; anaplastic	2830 (7.17)	507 (15.99)	
	Unknown	10508 (26.62)	1852 (58.40)	
	Well differentiated	3359 (8.51)	28 (0.88)	

Laterality (%)	Left	19407 (49.16)	1661 (52.38)	<0.0001
	Other	59 (0.15)	28 (0.88)	
	Right	20013 (50.69)	1482 (46.74)	

Pathological (%)	8120/3	1005 (2.55)	137 (4.32)	<0.0001
	8130/3	1008 (2.55)	25 (0.79)	
	8260/3	5169 (13.09)	109 (3.44)	
	8310/3	21172 (53.63)	1444 (45.54)	
	8312/3	6787 (17.19)	1036 (32.67)	
	8317/3	2213 (5.61)	18 (0.57)	
	Other (*n* < 1000)	2125 (5.38)	402 (12.68)	

*T* (%)	*T*1	27359 (69.30)	539 (17.00)	<0.0001
	*T*2	3656 (9.26)	591 (18.64)	
	*T*3	7164 (18.15)	1264 (39.86)	
	*T*4	732 (1.85)	411 (12.96)	
	*T*X	568 (1.44)	366 (11.54)	

*N* (%)	*N*0	36669 (92.88)	1719 (54.21)	<0.0001
	*N*1	1374 (3.48)	1057 (33.33)	
	*N*2	161 (0.41)	38 (1.20)	
	*N*X	1275 (3.23)	357 (11.26)	

Tumor size (median [IQR])	*N*A	40.000 (25.000, 60.000)	87.000 (62.000, 115.000)	<0.0001

Bone metastases (%)	No	38533 (97.60)	2166 (68.31)	<0.0001
	Yes	946 (2.40)	1005 (31.69)	

Brain metastases (%)	No	39301 (99.55)	2757 (86.94)	<0.0001
	Unknown	9 (0.02)	43 (1.36)	
	Yes	169 (0.43)	371 (11.70)	

Liver metastasis (%)	No	39061 (98.94)	2472 (77.96)	<0.0001
	Unknown	17 (0.04)	55 (1.73)	
	Yes	401 (1.02)	644 (20.31)	

**Table 2:** Univariate and multivariate logistic regression for patients with lung metastasis of renal cancer. It is reproduced from that article in the below format Table 2 is reproduced from Li et al. 2022 (under the Creative Commons (attribution license/public domain).

Characteristics	Univariate logistics			Multivariable logistics		
	OR	CI	*p*	OR	CI	*p*
Bone metastasis						
No	Ref	Ref	Ref	Ref	Ref	Ref
Yes	18.9	17.12–20.86	<0.001	4.83	4.27–5.46	<0.001
Brain metastasis						
No	Ref	Ref	Ref	Ref	Ref	Ref
Yes	31.29	25.98–37.69	<0.001	8.41	6.72–10.51	<0.001
Unknown	68.11	33.17–139.85	<0.001	6.13	2.35–15.98	<0.001
Grade						
Well differentiated	Ref	Ref	Ref	Ref	Ref	Ref
Moderately differentiated	1.98	1.34–2.94	0.001	1.41	0.94–2.11	0.102
Poorly differentiated	7.83	5.34–11.47	<0.001	2.71	1.82–4.04	<0.001
Undifferentiated; anaplastic	21.49	14.64–31.55	<0.001	4.58	3.05–6.87	<0.001
Unknown	21.14	14.53–30.77	<0.001	6.34	4.29–9.37	<0.001
Liver metastasis						
No	Ref	Ref	Ref	Ref	Ref	Ref
Yes	25.38	22.26–28.93	<0.001	4.23	3.6–4.96	<0.001
Unknown	51.12	29.63–88.2	<0.001	6.36	3.06–13.21	<0.001
*N*						
*N*0	Ref	Ref	Ref	Ref	Ref	Ref
*N*1	16.41	14.94–18.02	<0.001	3.79	3.37–4.25	<0.001
*N*2	5.03	3.52–7.19	<0.001	3.54	2.21–5.69	<0.001
*N*X	5.97	5.26–6.78	<0.001	2.33	1.96–2.77	<0.001
Primary site						
C64.9-Kidney	Ref	Ref	Ref	Ref	Ref	Ref
C65.9-Renal pelvis	0.81	0.68–0.98	0.027	0.38	0.3–0.49	<0.001
Sequence number						
One primary only	Ref	Ref	Ref	Ref	Ref	Ref
More	0.46	0.42–0.5	<0.001	0.62	0.56–0.69	<0.001
*T*						
*T*1	Ref	Ref	Ref	Ref	Ref	Ref
*T*2	8.21	7.26–9.27	<0.001	3.42	2.93–4	<0.001
*T*3	8.96	8.07–9.94	<0.001	4.44	3.89–5.07	<0.001
*T*4	28.5	24.58–33.04	<0.001	5.39	4.42–6.57	<0.001
*T*X	32.71	27.97–38.25	<0.001	5.67	4.63–6.94	<0.001
Tumor size	1.02	1.02–1.02	<0.001	1.01	1–1.01	<0.001

## Data Availability

The data used in this study are available from the corresponding author upon request.

## References

[1] Hua K. C., Hu Y. C. (2020). Establishment of predictive model for patients with kidney cancer bone metastasis: a study based on SEER database. *Transl Androl Urol*.

[2] Peired A. J., Campi R., Angelotti M. L. (2021). Sex and gender differences in kidney cancer: clinical and experimental evidence. *Cancers (Basel)*.

[3] Tanji N., Yokoyama M. (2011). Treatment of metastatic renal cell carcinoma and renal pelvic cancer. *Clinical and Experimental Nephrology*.

[4] Xiao R., Liu C., He W., Ma L. (2021). Prognostic factors and a nomogram predicting overall survival and cancer-specific survival for patients with collecting duct renal cell carcinoma. *BioMed Research International*.

[5] Ged Y., Voss M. H. (2021). Novel emerging biomarkers to immunotherapy in kidney cancer. *Therupatic Advances in Medical Oncology*.

[6] Chung C. (2020). From oxygen sensing to angiogenesis: Targeting the hypoxia signaling pathway in metastatic kidney cancer. *American Journal of Health-System Pharmacy*.

[7] Xue J., Chen W., Xu W. (2021). Patterns of distant metastases in patients with clear cell renal cell carcinoma-apopulation-based analysis. *Cancer Medicine*.

[8] Nishida J., Momoi Y., Miyakuni K. (2020). Epigenetic remodelling shapes inflammatory renal cancer and neutrophil-dependent metastasis. *Natural Cell Biology*.

[9] Zhang G., Zhang L., Sun S., Chen M. (2021). Identification of a novel defined immune-autophagy-related gene signature associated with clinical and prognostic features of kidney renal clear cell carcinoma. *Frontiers in Molecular Bioscience*.

[10] Marona P., Gorka J., Mazurek Z. (2017). MCPIP1 downregulation in clear cell renal cell carcinoma promotes vascularization and metastatic progression. *Cancer Research*.

[11] Lei Q. Q., Huang Y., Li B., Han L., Lv C. (2021). MiR-155-5p promotes metastasis and epithelial-mesenchymal transition of renal cell carcinoma by targeting apoptosis-inducing factor. *International Journal Biology Markers*.

[12] Abdel-Rahman O. (2017). Clinical correlates and prognostic value of different metastatic sites in metastatic renal cell carcinoma. *Future Oncology*.

[13] Shinder B. M., Rhee K., Farrell D. (2017). Surgical management of advanced and metastatic renal cell carcinoma: a multidisciplinary approach. *Frontiers Oncology*.

[14] Park J. Y., Tae B. S., Jeong C. W. (2020). Development of the clinical calculator for mortality of patients with metastatic clear cell type renal cell carcinoma: an analysis of patients from korean renal cancer study group database. *Investigate Clinical Urology*.

[15] Danno T., Iwata S., Niimi F., Honda S., Okada H., Azuma T. (2021). Nivolumab and ipilimumab combination immunotherapy for patients with metastatic collecting duct carcinoma. *Case Reports in Urology*.

[16] Schulz S., Woerl A. C., Jungmann F. (2021). Multimodal deep learning for prognosis prediction in renal cancer. *Frontiers Oncology*.

[17] Lee M., Wei S., Anaokar J., Uzzo R., Kutikov A. (2021). Kidney cancer management 3.0: can artificial intelligence make us better?. *Current Opinion in Urology*.

[18] Xi W., Hou Y., Hu X. (2021). Prognostic significance of pseudocapsule status in patients with metastatic renal cell carcinoma treated with tyrosine kinase inhibitors. *Translational Andrology and Urology*.

[19] Dong S., Yang H., Tang Z. R. (2021). Development and validation of a predictive model to evaluate the risk of bone metastasis in kidney cancer. *Frontiers in Oncology*.

[20] Sheng X., Lu X., Wu J., Chen L., Cao H. (2021). A nomogram predicting the prognosis of renal cell carcinoma patients with lung metastases. *BioMed Research International*.

[21] Zisman A., Pantuck A. J., Dorey F. (2001). Improved prognostication of renal cell carcinoma using an integrated staging system. *Journal of Clinical Oncology*.

[22] Volpe A., Bollito E., Bozzola C. (2016). Classification of histologic patterns of pseudocapsular invasion in organ-confined renal cell carcinoma. *Clinical Genitourinary Cancer*.

[23] Xi W., Wang J., Liu L. (2018). Evaluation of tumor pseudocapsule status and its prognostic significance in renal cell carcinoma. *Journal of Urology*.

[24] Bridgeman V. L., Vermeulen P. B., Foo S. (2017). Vessel co-option is common in human lung metastases and mediates resistance to anti-angiogenic therapy in preclinical lung metastasis models. *Journal of Pathology*.

[25] Wehrli N. E., Kim M. J., Matza B. W., Melamed J., Taneja S. S., Rosenkrantz A. B. (2013). Utility of MRI features in differentiation of central renal cell carcinoma and renal pelvic urothelial carcinoma. *American Journal of Roentgenology*.

[26] Petrozza V., Costantini M., Tito C. (2020). Emerging role of secreted miR-210-3p as potential biomarker for clear cell renal cell carcinoma metastasis. *Cancer Biomarkers*.

[27] Pecoraro A., Palumbo C., Knipper S. (2020). Histologic Subtype, Tumor Grade, Tumor Size, and Race Can Accurately Predict the Probability of Synchronous Metastases in T2 Renal Cell Carcinoma. *Clinical Genitourinary Cancer*.

